# “I want to live life, not just be in it!”: a qualitative study on existential aspects of choosing to reduce or stop psychotropic medication

**DOI:** 10.1080/17482631.2025.2572509

**Published:** 2025-10-16

**Authors:** Stine Madsen Kvaløy, Oddgeir Synnes, Anne Austad

**Affiliations:** Centre for Diaconia and Professional Practice, VID Specialized University, Oslo, Norway

**Keywords:** Medication-free treatment, psychotropic medication tapering, lived experience, existential meaning-making, spirituality, existential health

## Abstract

**Purpose:**

While psychotropic medication is commonly used to treat severe mental illnesses, some patients perceive it as a barrier to meaningful aspects of their lives, motivating them to reduce or stop taking it. This qualitative study aims to contribute to the existential understanding of the choice to taper psychotropic medication.

**Methods:**

An existential phenomenological method was employed, investigating and analyzing the lived experiences of 15 individuals in Norway in relation to their choice to taper their psychotropic medication with professional support.

**Results:**

A drive towards existential health was identified, expressed through four types of quests: 1) the quest for a meaningful daily life, 2) the quest for a true self, 3) the quest for a deep sense of belonging with others, and 4) the quest to integrate spirituality in life.

**Conclusions:**

This study indicates that the choice to reduce or stop taking psychotropic medication may encompass complex, existential dimensions beyond symptom management, affecting fundamental life aspects. For mental health professionals, these findings emphasize the importance of understanding these dimensions to better support individuals diagnosed with severe mental illness in their medication decisions and tapering processes.

## Introduction

### Background

Psychotropic medication serves as an umbrella term for a range of drugs designed to treat mental illnesses by altering thoughts, moods, behaviours, and perception. It includes antidepressants, antipsychotics, mood stabilizers, stimulants, anxiolytics, and sedatives. The use of psychotropic medication in the treatment of mental illnesses is prevalent and continues to rise (Brauer et al., 2021).

While many individuals using psychotropic medications report satisfaction with their effects, significant side effects have been documented (Keltner & Faulks, 2005; Moncrieff et al., 2009). When prescribing psychotropics, clinicians will always have to weigh the desired effects against unwanted side effects (Murray et al., 2016; Yeisen et al., 2017). For instance, while antipsychotics may reduce symptoms such as hallucinations and delusions, adverse effects such as drowsiness, fatigue, sedation, sexual dysfunction, loss of motivation, slowed cognitive processing, emotional numbness, and suicidal thoughts significantly contribute to the desire to taper medication in a large number of individuals (Bjornestad et al., 2020; Read & Williams, 2019; Semahegn et al., 2020).

Commonly reported experiences, particularly in relation to antipsychotics, include “feeling like a zombie” and experiencing a sense of losing one's sense of self (Asher et al., 2023; Awad, 1993; Bentley, 2010; Conneely et al., 2025; Moncrieff et al., 2009; Tandon et al., 2020; Thompson et al., 2020). In addition, some studies have found that the use of psychotropic medications can interfere with existential experiences such as meaning in life (Bentley, 2010; Carrick, 2004; Vanderpot et al., 2018) and spirituality (Borras et al., 2007; Hagen et al., 2010; Mohr et al., 2006; Vanderpot et al., 2018).

Perceiving medication as an impediment to meaningful existential aspects of patients' lives has been found to motivate them to consider reducing or stopping it (Blindheim, 2020; Hagen et al., 2010; Hvite Ørn [White Eagle], 2019; Seeman & Seeman, 2012; Standal & Heiervang, 2018). However, the potential interference of medication with existential aspects of an individual's experience does not inherently render pharmacological interventions negative. Research also indicates that medications can serve as important recovery tools, which individuals can actively employ to enhance their overall well-being and meaning in life (Baker et al., 2013; Carrick et al., 2004; Moncrieff et al., 2009; Vanderpot et al., 2018).

### The existential dimension of health

According to the World Health Organisation (WHO), “a mental disorder is characterized by a clinically significant disturbance in an individual's cognition, emotional regulation, or behaviour. It is usually associated with distress or impairment in important areas of functioning” (WHO, 2022). Mental illness has also been acknowledged to interact with existential issues such as meaning, values, hope, faith, and the essence of being human (Bentall, 2004; Huguelet & Koenig, 2009; Pargament, 2007; Paloutzian & Park, 2013; Shafranske, 1996; Yalom, 1980).

To comprehend the term “existential,” it is essential to briefly explore its philosophical underpinnings. Existentialist philosophy centres on “being in the world” and lived experiences as fundamental to understanding humanity (Heidegger, 1927, 1949; Merleau-Ponty, 2012/1945). Themes such as authenticity are often linked to the notion of living in accordance with one's true self, often at odds with societal norms (Heidegger, 1927, 1949; Sartre, 1943). Common existential questions pertain to meaning in life, identity, our purpose, how we relate to death and suffering, and the existence of higher powers (Frankl, 2004/1946; Heidegger, 1927, 1949; Kierkegaard, 1971/1843; Nietzsche, 1998/1886; Sartre, 1943).

Related to Yalom’s (1980) four ultimate concerns of human existence—isolation, death, meaninglessness, and freedom—several Scandinavian scholars propose using “existential” as an umbrella term to describe meaning-making responses to life's inherent challenges, whether situated within religious, spiritual, or secular contexts (DeMarinis, 2008; LaCour & Hvidt, 2010; Lloyd et al., 2017; Nygaard et al., 2022; Stifoss-Hanssen, 1999). In addition, the concept of “existential health” has recently emerged in Scandinavia, to represent an individual's relation to their own health (Binder, 2022; LaCour, 2025; Sigurdson, 2016, 2019). Distinct from “spiritual health,” which is often seen to encompass relationships with transcendent aspects of existence, existential health integrates secular worldviews and emphasizes subjective experiences of illness and health. Good existential health refers to the capability to relate authentically and meaningfully to illness and suffering—whether physical, mental, social, or spiritual.

Existential meaning-making has been shown to be of particular importance for individuals diagnosed with schizophrenia (Borras et al., 2007; Danbolt et al., 2011; Mohr et al., 2006, 2012), bipolar disorder (Mitchell & Romans, 2003), and major depression (Stålsett, 2012). Research indicates that individuals with severe mental illness derive benefits from incorporating existential meaning-making dimensions into their treatment (Frøkedal & Austad, 2019; Holm et al., 2024a, 2024b; Koslander et al., 2020; Trapman & Braam, 2023). Furthermore, existential frameworks have proven effective in assisting individuals with severe treatment-resistant mental illnesses in achieving freedom from psychotropic drugs and significant post-treatment functionality (Hammer et al., 2018).

The World Health Organisation (WHO) stresses the importance of existential concerns in mental health care when advocating for a recovery focus “helping people to regain control of their identity and life, have hope for the future, and to live a life that has meaning for them, whether that be through work, relationships, community engagement, spirituality or some or all of these.” (World Health Organisation, 2019, p. xi). Similarly, in Norway, where this study is situated, Helsedirektoratet [The Norwegian Directorate of Health] recognizes that “mental disorders fundamentally affect existential questions” and that “necessary consideration must be given to man's spiritual and cultural needs, not just the biological and social ones” (Helsedirektoratet [The Norwegian Directorate of Health], 2013, section 3.1).

### Psychotropic medication and tapering

In addition to emphasising the importance of meaning-making in mental health care, the WHO highlights that biomedical treatments for mental disorders should not be considered the sole therapeutic option (WHO, 2022). Adverse effects of medication are reportedly driving many individuals to want to stop taking their medication (Bjornestad et al., 2020; Read & Williams, 2019; Semahegn et al., 2020; Standal et al., 2021), and many individuals prescribed psychotropic medications ultimately choose to discontinue their use and seek support in this process (Watts et al., 2021). Tapering—a gradual reduction or stopping of medication—is recognized to be the most appropriate method for discontinuation (Boland et al., 2024). In Norway, a medication-free treatment (MFT) option became available in 2015, providing individuals with severe mental illness the opportunity to taper their medication with professional support. The established MFT units typically adopt a recovery-oriented approach (Slade et al., 2008), providing inpatient and open ward services that prioritize individuals diagnosed with severe mental illness (Standal et al., 2021, 2024).

Medication tapering is administered variably across MFT units nationwide, with each facility offering tailored plans to meet individual patient needs. Generally, these units emphasize a gradual tapering process, with some hospitals providing extended stays of several months for comprehensive tapering, while others allow for multiple planned admissions over several years. The tapering process is individualized, allowing for pauses when necessary. Both pharmacological and therapeutic interventions are provided to help manage symptoms throughout this process, tailored to meet the unique needs of each individual. In addition to tapering support, MFT units incorporate a range of therapeutic activities, including creative arts and music therapy, as well as individual and group therapy sessions. Furthermore, some actively involve patients' networks in the recovery process to enhance support and engagement.

MFT staff accounts indicate a strong emphasis on prioritising patients' values, fostering their sense of responsibility for their own treatment and recovery (Beyene et al., 2023; Oedegaard et al., 2022a, 2022b; Reitan et al., 2024b; Standal et al., 2024). Some studies evaluating MFT have identified existential themes such as meaning, hope, freedom, and belonging as integral to patients' decision-making processes regarding tapering (Oedegaard et al., 2020; Reitan et al., 2024a, 2024c; Standal et al., 2021). However, these are not studies that investigate these aspects in depth through an existential theoretical lens, and more research is needed to establish how these dimensions can inform patients' choices regarding tapering.

### Aim of study

By neglecting to consider how medication use may interact with existential concerns and motivate decisions regarding reducing or stopping, one might inadvertently overlook the complex realities faced by patients. Specifically, lived experience research is essential to gain deeper insights into the ways in which existential concerns shape individual experiences and decisions, ultimately guiding more personalized and supportive tapering and recovery plans.

Thus, this qualitative study aims to enrich the understanding of the existential aspects related to the decision to taper psychotropic medication by exploring the lived experiences of individuals in Norway who have made this choice. The research question is: how are existential aspects experienced through the choice to taper psychotropic medication?

## Method

### Design

An existential phenomenological approach was chosen because of its capacity to grasp lived experiences and detect deeper meanings (Churchill, 2021). The goal was not to find verifiable “truths” but providing an understanding of how existential aspects may play out for the participants in their choice to taper their medication. This involved embracing openness to what could unfold during the interview process. It requires the researcher to set aside preconceived notions and judgments, placing them in “brackets” as Husserl (1913) would call it, and truly listening to what the participant is conveying. By doing so, the researcher endeavours to uncover not only the immediate concerns, but also what truly matters to the individual in their existence.

To grasp the lifeworld of a participant, one must engage empathically, allowing the complexities of their experiences to emerge organically. As researchers, we must approach the investigation experience with curiosity and willingness to be surprised, letting go of what we think we know about a phenomenon. In this way, we created space for genuine understanding, unveiling the richness of the participants’ journeys and the existential significance of their choices. This attunement to an individual's voice illuminates the fundamental aspects of their existence, revealing insights into what is vital to them in their lives (Churchill, 2021).

### Setting

Our study consisted of in-depth, semi-structured interviews with 15 former patients of MFT in Norway. At the time of data collection, four different hospitals in Norway offered inpatient treatment in wards solely devoted to the MFT option. The current study included two of them: one in the southeast region of the country and one in the north. One of the hospitals was a state hospital, whereas the other was a private hospital, where patients by referral got the cost covered by the government. The private hospital offered stays of four months with gradual tapering, while the state hospital provided multiple planned stays tailored to individual needs, enabling a slow tapering process that could extend over several years in some cases.

### Participants

The participants were invited to participate in the study through invitations sent out by the cooperating MFT units. These units also promoted the research on social media (Facebook). The researcher who managed participant administration was unaware of the identities of individuals invited to join the study until they expressed interest in participating. The inclusion criteria were as follows: 1) experience of being diagnosed with severe mental illness, 2) experience taking psychotropic medication, 3) former patient at one of the participating hospitals in the study, with experience of one or more attempts to taper psychotropic medication, and 4) openness to talk about existential themes.

All individuals who expressed interest were eligible to participate. Eight women and seven men took part, age range 22−64. Regarding the types of diagnosis and medication, this information was not formally collected but derived from the interview transcriptions where the participants chose to enclose it. According to the information the participants chose to provide in the interviews, most participants had taken antipsychotics and/or mood stabilizers for three months or more. Some had also taken stimulants, anxiolytics, and sedatives. A few also reported experiences with antidepressants. Most participants had been taking medication regularly for several years. Some of the participants had prior experience in independently reducing or discontinuing their medication before being admitted to the MFT. This included both abrupt cessation and gradual tapering. Following their admission to the MFT, they were given personalized tapering plans tailored to their individual needs, which varied from a gradual tapering process over the course of up to four months with professional support, to an even slower reduction involving several planned hospitalisations. At the point of interviewing, 11 participants were still taking some amount of medication, whereas four had stopped completely.

### Data collection and ethical considerations

A semi-structured interview guide was used. Participants were asked about their relationship with medication, their reasons for wanting to reduce or stop, their tapering experiences, their relationship with MFT and why they chose it, what they associated with the term “existential,” what worldview they considered themselves to have, what they found important and meaningful in their lives, and how medication use and tapering might influence these aspects. The questions were intended to be opening up rather than directly inquiring about the research question. The participants were encouraged to talk freely about their experiences.

The first author conducted the interviews. The interviews were conducted either face-to-face or digitally, depending on which format the participant preferred and found most suitable. They took place between Fall 2021 and Summer 2022 and lasted between 45 and 150 min, with most lasting approximately 90 min. Some interviews were divided into several parts to accommodate participants' needs.

Expenses were covered. Participants were contacted 1−2 days after each interview to provide an opportunity for subsequent reflections and questions and to ensure that they had not suffered any harm. None of the participants were considered to have suffered harm. Participants signed a written informed consent form prior to participation and were given the opportunity to withdraw from the study at any point without explanation. The interviews were audio recorded and transcribed. We ensured that no data in the transcriptions could reveal the identity of any participant, and all the names in this article are pseudonyms. The interviews were conducted in Norwegian, and the analysis was also performed in Norwegian. Quotes selected for inclusion in the article were translated into English by the first author, with support from AI language assistance.

The study was conducted in accordance with the Declaration of Helsinki. As per Norwegian laws, it was approved by the Norwegian Regional Committee for Ethics and Research (REK vest, 218108) and the Norwegian Agency for Shared Services in Education and Research (SIKT 694899).

### Analysis

The analysis was guided by Churchill's (2021) existential phenomenological method for approaching data. Given that the study focused on existential themes and medication tapering, its primary goal was not to determine whether existential elements were present or not, but rather to explore how the choice to taper medication can be understood from an existential perspective. While certain existential themes structured the interview approach, the analysis remained grounded in the participants' own framing of their experiences. Churchill (ibid) recommends a mode of deep listening for the participants’ “intentionality,” which “points to the meaning-making acts on the part of the participant during the experienced situation described in their data” (p. 29). The goal of the analysis was to find common ways across the sample for participants to relate to possible existential issues.

The first author stood for the initial part of the analysis. Excessive notes were taken during this process. After the first phase, the initial familiarisation with the data, the analysis went through different phases of formal analysis. First, a sense of the whole was achieved through reading and reflection. The data were then divided into initial meaning units. Following this, more transformative reflections were carried out, in which the first author engaged in the meaning units in a more interpretive manner.

Awareness of pre-understanding was constantly worked on and challenged through notetaking, re-reading, and writing out possible understandings, while discussing these with the other authors. In this process, the first author employed an attitude of “empathic dwelling” (Churchill, ibid, p. 53), where the researcher engages with the material in a slow and empathic way, using her own senses and intuition to take thoroughly onboard the descriptions of the participants' experiences, deeply detecting what meaning units that defines their lived experiences. These meanings were then discussed with the other authors and categorized, first individually and then across the dataset, resulting in a final structural description of the interpreted meanings.

### Reflection around pre-understanding

The first author has a BSc (Hons) in Psychology, an MA in Psychoanalytic Studies, and is a trained transpersonal psychotherapist. With experience in mental health outreach work, psychiatric wards, and therapeutic practice, she is skilled in empathic handling of diverse mental health issues, emphasising meaning in treatment. Her training in the transpersonal tradition allowed her to explore underlying existential issues. This perspective led to deeper insights but also required awareness of potential preconceptions around existential elements. Her experience aided in navigating the complex dynamics and understanding the lived experiences and intentions of the participants. This ability to seek deeper meanings is valuable for existential phenomenological research, both in the interview process and in order to get “under the data” during the analysis (Churchill, 2021).

The second author, with a background in literature and health humanities, and the third author, with a background in theology and psychology of religion, contributed to critically discussing the meaning units.

## Results

When asked about their motivations for choosing to reduce or stop taking medication, the participants were eager to share their experiences with medication and the mental health system. They recounted various processes -often multiple attempts- of trying to discontinue their medication, employing a range of methods from abrupt cessation to planned gradual tapering, either independently or with professional guidance. Although the primary focus of the interviews was the choice to taper with MFT, participants frequently included other relevant experiences. Additionally, they noted how their relationships with medication had evolved over time. The authors interpreted these narratives as playing a crucial role in understanding the participants' lived experiences and motivations for choosing medication tapering. The participants' narratives were divided into four categories: daily functioning, sense of self, social domains, and spirituality.

The authors interpreted the narratives as transcending mere stories from beginning to end; they conveyed a sense of purpose that the participants were striving towards. Recognising that these narratives encompassed ongoing journeys toward something profound, we chose to refer to them as quests. We found that the participants juggle different aspects of their existence, in which their choices regarding medication play an important role. On deeper examination of these existential aspects, it appeared that the participants, in their choice to taper medication through MFT, were engaged in four different existential quests: 1) the quest for a meaningful daily life; 2) the quest for a true self; 3) the quest for a deep sense of belonging with others; and 4) the quest to integrate spirituality in their lives.

### The quest for a meaningful everyday life

This category concerns how the choice of medication tapering entails searching for a meaningful everyday life. The participants told stories about struggling to maintain daily functioning due to the side effects of medication, which drove them into tapering attempts. Weight gain, sleepiness, chronic diarrhoea, confusion, dizziness, being cut off from feelings, loss of will-power, and worsening of symptoms were among the most common side effects mentioned. Being present with children, keeping in contact with family and friends, maintaining good physical health, engaging in meaningful activities, and hobbies were valuable aspects of life lost to side effects. As a cost of quieting symptoms, joy and meaning disappeared, taking away essential elements of a good life:

*After starting medication, my joy faded away as I faced severe side effects from [mood stabilizer] that affected my digestion and caused chronic diarrhoea. For nine years, finding a restroom was my constant concern, profoundly impacting my quality of life. -Mia*

According to many participants, medication was initially prescribed to reduce the symptoms of mental illness, often in acute phases of distress. Although medication has aided certain aspects in many cases, such as stopping suicidal behaviour or getting rid of unwanted hallucinations, ruminations, anxiety, or insomnia, in the long run, it had prevented them from functioning in daily life. Some participants also found that suicidal thoughts worsened while on medication. For many of the participants, being on medication became a question of value; having to decide if getting rid of symptoms was more important than aspects of life that they lost to side effects. Realising that the aspects lost to side effects held crucial elements for feeling alive, it overshadowed peace from symptoms for some. The attempt to downsize became an existential quest to claim life back, being alive rather than just existing:

*There and then I felt medication was necessary for my head to relax. But also, my head got completely blank. All my willpower disappeared; I didn’t feel like doing anything. In the end I decided I wanted to live, not just be in life. To me, living life is about relations and belonging. To sit in an apartment on your own is not a life. -Jacob*

The side effects not only had a profound impact on quality of life but also affected the fundamental experience of living. Many reported feeling a sense of disconnection from life:

*You are not a human. You can take something to sleep at night, but then you walk around in a coma all day. I’d rather sleep badly and be present in everyday life. Those who are on antipsychotics are not present in this world. It’s a veil or something, I don’t know how to describe it. -Leah*

A common description was the notion or metaphor of “zombie,” which was experienced as a direct side effect of antipsychotics especially:

*It went to hell. The problem was that I walked around like a living zombie, because the side effects were so strong. -Simon*

*One becomes chemically lobotomized; I have personally been in a zombie state, more like a living dead than a living person. -David*

Nevertheless, several participants indicated that fully discontinuing their medication did not resolve the issue; instead, they found that troublesome symptoms returned and carried a heavy burden, rendering life feel meaningless once more. Like Mia describes it:

*It became darker and darker, and I believe I would have been able to stop it if I had medication. I felt like I was back at square one because I had stopped taking [mood stabilizer]. -Mia*

For Mia and some other participants, the time after deprescribing and being discharged from the MFT entailed experiencing such a degree of unwanted symptoms that they needed treatment in ordinary inpatient mental health care. Many learned that they needed some form of medication, but it was more about finding the right type of medication, the right dosage, and ways of usage in accordance with meaning in their lives:

*I’m afraid people will judge me [for taking medication], but then again… I can say that I’m functioning. I must take medication, but I function. I’m not running around completely mad and spending money I don’t have and so on. I have a job, and I’m holding on to it. That feeling is good. That’s meaning in life to me! I manage, I’m not an idiot, I manage life like everyone else. -Thomas*

Others wanted to stay completely off medication, but continued to search for other types of therapy to support them in that choice. Some described their experience with tapering through MFT as unsuccessful and were disappointed, others reported that it nevertheless had gotten them further in realising what was meaningful to them. Although not all participants had landed in a place where they were content with medication use, treatment, and symptoms at the time of the interview, they all described ongoing processes of finding one’s way through it. Central to these processes was to find a balance with the common pursuit of meaning in everyday life.

### The quest for a true self

This category is about the quest to live authentically in accordance with one's true self while reflecting on the role of medications in that. The participants used words like “true,” “real” and “essence” when talking about themselves. In our interpretation, these expressions had the commonality of holding an existential meaning of a true or authentic self, and the participants described how they perceived medication to interfere with it:

*Medication for me has been a pressure cap holding down who I really am. I became very reduced in my vitality. Maybe not suicidal, but “I only exist in suffering”, I remember thinking. “I am a person who lives in suffering, and I accept that”. Because that's how it was, because the medicines were supposed to help me. -Oliver*

Medication influenced many of the participants' sense of self, which fuelled their wish to taper. This could be in the form of pharmaceutical side effects, stopping their true self from prospering in some way:

*[On medication] I didn’t have any feelings for my kids. None for my partner. But I knew that I loved them. I didn’t have any feelings; I was indifferent, careless and cut off. Trapped in myself. Neither happy nor sad. Just stuck, standing still at the same point. -Hannah*

or shutting down what was experienced as the essence of who they were:

*With [mood stabilizer], I just felt like bleh. You completely lose your identity. I don’t want a life without being me. People say if you take [mood stabilizer] you can live a good life, but I disagree. Yes, you can live a comfortable life for others because you are just a dot. I want to be myself and the essence of who I am. -Eva*

Taking medication could also be a symbolic act that troubled the participants in their relationships with their true selves. Some reported feeling faulty or impure on medication. For many, the notion of the true self was associated with the unmedicated self:

*I cannot explain why I do not like medications. It might have something to do with the fact that I do not want to be influenced by anything external. I believe those who manage without medications do better than those who take medications. They find a solution that is healthier and more correct. They find a truth or meaning in life. -John*

In some instances, although medication initially had been perceived as the main interference with their true self and driven them to taper, some participants later found their medicated self to be the most real. By adjusting dosages or switching medications, they were better able to manage symptoms that were not perceived as parts of themselves. In any event, the quest for a true self appeared to be prevalent in relation to medication and tapering choices across the sample.

### The quest for a deep sense of belonging with others

This category concerns interpersonal relationships and how participants conveyed a desire for profound connection, also perceiving themselves as integral components of a larger system beyond their individual selves. Relationships with other people were something that occupied all participants. Their desire to tamper with their medication was largely rooted in the challenging juggle of symptoms and side effects, which either made it difficult or easier to obtain and maintain close relationships. Like Catherine described her disconnection from others while on medication:

*My back was curved, I was drooling all the time and slept 18−19 hours a day because of side-effects. I had work practice for two hours a day where I basically just sat on a chair watching the others. I was completely paralysed and cut off. No joy or sorrow. And my son grew up in all this! Choosing downsizing through MFT meant giving myself permission to live. -Catherine*

For most participants, the side effect of being cut off from emotions was the biggest difficulty when it came to medication use and relationships. Not feeling love for close ones could be difficult:

*I don’t really want to tell my mother or sister that I’m not feeling any love for them when they’re near me. That’s something that can really hurt, and I don’t think it’s bringing me any closer to people. For me, it’s something essential that’s been taken away. -Victoria*

At the same time, many reported needing medications in order to function in relationships -like keeping tempers, looking after children, hindering symptoms in stopping them from taking part in social activities, and similar:

*If I didn’t have my daughter, I could try [to come off medication] once more. But I don’t, because of her. I must have a place to live, and I need an income. If I don’t manage to keep to my routine, my life will fall apart. And then I won’t have anything to live for anymore, and everything will be pointless. -Julia*

Belonging was a powerful influence that often surpassed considerations, such as minor side effects or personal growth. Belonging could also be on a larger scale than one-to-one relationships; it encompassed the desire to connect with society, to find one's place, and to be part of a group.

### The quest to integrate spirituality in life

This category entails the spiritual aspects associated with medication use and how it is linked to the choice to taper. More than half of the participants highlighted spirituality as a significant part of their lives, discussing concepts of the transcendent. Most of those who mentioned spirituality expressed that their medications interfered with it. It was commonly recognized that medication, particularly antipsychotics and mood-stabilizers, placed a lid on spiritual experiences. Therefore, choosing to taper involved lifting the lid. For some, it could mean both negative and positive aspects of spirituality that medication had held at bay:

*Religion and the existential questions have been a big part of my upbringing. My whole life has been about faith and God. The medication puts a lid on all that. When I try to downsize, it all comes back. -Emma*

For others, the actual tapering process could be experienced as a form of awakening, which could be both frightening and enlightening:

*It [the drug] was quite heavy and I was on a low dose compared to the maximum dose. But when I was completely clean, it was crazy, because I didn't understand what was coming up. It was a personality or soul or whatever that had been suppressed for a long time that blossomed, and it was scary. Almost a spiritual experience. -Oliver*

It appeared to be a common experience that medication numbed spiritual experiences. Because of the effect of medication on spirituality, the choice of whether to take it became a difficult and ongoing juggle for many participants. They continually evaluated the impact and importance of spirituality in their lives, and many reported ambivalences in wanting to retain some parts of their spirituality, but not all of them. Many reported challenges in understanding their spirituality and incorporating it in ways that were fruitful, with the aim of not throwing the baby out with the bathwater by medicating away positive aspects. Some found this too hard and ended up discarding spirituality in favour of other valuable aspects, such as daily functioning or relationships:

*Now I’m 43 and I’ve been on medication since I was 18, so I hardly know life without medication. I believe it could be a healing process for me to quit all medication, maybe go on a journey through the chakra system. But the thing is, I’m a family father, and a chakra journey is powerful. I believe there are strong forces, and one loses oneself. It requires competent people who are not easily thrown to assist. Right now, my life will not handle it. -David*

Religious and spiritual images, themes, and figures were prominent. This was in some cases interpreted as symptoms of mental illness, for others, a natural part of life. About one-third of the participants reported extraordinary spiritual experiences, such as communication with the deceased or receiving messages from God. Some noted that these experiences were more intense with less medication, while others noted that medication had little impact. Although medication did not necessarily hinder all spiritual experiences, it seemed to complicate them for some. One woman noted that medication interfered with her healing abilities, making it difficult for her to differentiate between various elements. She chose to refrain from practicing healing while on medication, as she considered it unclean and unethical to practice under such influence.

Additionally, some participants not particularly concerned with spirituality spoke of existential pondering and experiences of understanding the meaning of the universe, which were stronger when they were not on medication. Some interpreted this as a symptom of mental illness, whereas others were open to the possibility of a higher meaning. Nevertheless, as with participants who explicitly spoke of spirituality, they all had a common goal to make sense of their experiences and integrate them in ways they could live contently with. This could include using medication to keep their experiences at bay or tapering them to embrace more explorative journeys. Those who chose to embrace spirituality reported that it was an important factor in their recovery process.

### Opposing and linked quests

The four categories of quests outlined above can be viewed independently, but they also interact with each other. They may sometimes oppose each other while being interconnected, thereby creating a dynamic interplay. The participants were constantly juggling different aspects of their lives and medication use. Choosing between two or more opposing aspects were common. For example, some people chose to be on medication to function in their daily lives with meaningful family obligations, rather than getting off medication and embarking on spiritual journeys or self-development towards a more authentic self.

However, different aspects were also linked and depended on each other. For example, relationships could interfere with the sense of self, and feelings of self-worth and confidence could reflect important interactions with friends and family. Furthermore, the degree of incorporation of spirituality could strengthen the sense of self and enhance meaning in life, making life worth living. Finding a balance between the quests was a large part of it, which sometimes involved finding the right amount of the right medication that would aid this balance. Those who reported having “a bit of all” and being to some degree in control of the juggles portrayed themselves as content with life. This could be experienced and expressed in paradoxical and unexpected ways:

*Despite being mentally weak, I am mentally strong. It’s something underneath that makes me strong. It seems strange to say that someone who suffers mentally is also strong. It’s a strange thing to think, but also something that is a little nice to think about. -John*

Overall, the juggling of various quests clearly indicated that the participants were strongly driven to seek “something more”—not just solutions in one or more aspects, but in a larger, more cohesive whole. This whole appeared to resonate on a profound existential level.

## Discussion

### The existential aspect of decision-making

In our study, participants reported side effects as one of the main reasons for tapering, which is in line with previous research (Bjornestad et al., 2020; Read & Williams, 2019; Semahegn et al., 2020; Standal et al., 2021). While it may seem straightforward to assess whether medication's side effects outweigh its benefits, our findings suggest that participants engage in a deeper evaluative process rooted in personal values and meaning. The choice of medication is not merely a “pros and cons” decision; rather, it is influenced by the individual's evolving values and priorities as well as what they find meaningful. While a third person (e.g., a clinician) might consider it most important to take control of symptoms, especially in acute phases, the values of the person taking the medication might differ or change over time. This resonates with previous research, which highlights how medications can interfere with fundamental aspects of leading a fulfilling life, prompting individuals to grapple with the question of whether the benefits are worth the associated costs (Bentley, 2010; Bjornstad et al., 2020; Flore et al., 2019; Hagen et al., 2010; Moncrieff et al., 2009; Reitan et al., 2024c).

Furthermore, the importance of autonomy in decision-making for pharmaceutical treatment has been well documented in research (Seeman & Seeman, 2012; Yeisen et al., 2017). It is understandable that individuals wish to be actively involved in their treatment choices, particularly given the significant impact that medications can have on their lives. In addition, many individuals who have been forced into pharmaceutical treatment may feel compelled to assert control by actively opposing it later on. However, based on our findings, we argue it is important that it does not get reduced to superficial discussions about empowerment and making patients feel heard. Such conversations risk becoming hollow if they do not adequately address the direct and profound impact that medications can have on individuals' lives. This was also recognized by Reitan et al. (2024c) when discussing the complexity of tapering through MFT.

### Claiming back the lived body

The “zombie” metaphor revealed in our study is well established in previous research (Asher et al., 2023; Awad, 1993; Bentley, 2010; Conneely et al., 2025; Moncrieff et al., 2009; Tandon et al., 2020; Thompson et al., 2020) and serves to illustrate a shared experience among individuals taking psychotropic medications, particularly antipsychotics.

Although not interpreted through the same existential lens, previous research on MFT patients' experiences includes metaphors of the zombie and how tapering involves getting the nervous system back on track and perceiving life as more meaningful (Reitan et al., 2024a, 2024b, 2024c; Standal et al., 2021). The need for a sense of coherence is also emphasized by Reitan et al. (2024a).

From an existential perspective, the zombie representation holds significant implications for understanding the lived experiences of those affected, where individuals feel more akin to the living dead than fully alive. The existential phenomenologist (Merleau-Ponty, 2012/1945) argued that our bodily presence is fundamental to experiencing phenomena, allowing the world to appear meaningful and coherent. We perceive the world through our senses, and this forms our subjective experience of our daily life. In our study, participants described how psychotropic medication directly impacts their sensing nervous system, their lived body, and alters their perception of the world. In this way, meaningful aspects are taken away. Finding ways to live dignified with symptoms becomes a way of responding to this distortion. With the goal of relating to suffering and symptoms as part of life, claiming back the lived body with its senses and perceptions becomes an existential quest.

### Exploring who I am in this world

Our findings indicate that medication influences participants' sense of self, which is well established in previous research (Thompson et al., 2020). However, the descriptions in our study extend beyond typical reflections of personality types, psychological ego-structures, and cultural identity factors. The type of self the participants appear to be longing for through tapering is articulated with terms such as “true,” “real” and “essence.” This is similar to the findings of Standal et al. (2021), who also found the unmedicated self to be associated with the “real self” among participants who chose the MFT at another unit in Norway. Further, Standal retrieved the notion of wanting to “live as the person I am” (Standal et al., ibid, p. 1654) in relation to the cautiousness of medication.

We argue that such pursuits ought to be viewed through an existential lens, as it aids in forming a fulfilling, genuine, and meaningful response to the question, “Who am I in this world?”. This question further delves into fundamental questions, such as “What does it mean to be human?,” “What is a self?” and even “What constitutes a fractured self?.” These existential inquiries lack definitive answers and it is the search for a broader meaning that fuels the quest. Our participants' choices around medication and tapering are guided by what brings them closest to what they perceive to be their true self. Although it could be side effects of medication that was perceived as a barrier to the true self, mental health symptoms could also alienate some individuals from themselves, making the quest for the true self a complicated matter. According to existentialist philosophy, the true and authentic self is not something inherently given, but rather something that each person must actively seek and renegotiate (Heidegger, 1927,^1949^; Sartre, 1943). Therefore, searching for the true self becomes an existential quest. In our sample, making sense of mental health symptoms, as well as own reactions to medication, appeared to be important aspects of this quest.

### No man is an island

Given that psychotropic drugs are well known to induce emotional detachment, apathy, and reduced initiative (Moncrieff et al., 2009), it is perhaps not surprising that the participants reported that their medication use affected their relationships with other people. From an existential perspective, we argue that while physical proximity to others may fulfil a superficial social need, deeper connections—those that foster genuine emotional engagement and mutual understanding—are essential for a meaningful life. Our findings underscore this: because participants perceived medication as a hindrance for emotional attachment and initiative in relations, it stopped them from deeply connecting with others. Because of their longing for this connection, medication became undesired. However, if being off medication meant not being able to function well alongside others, it changed the picture.

This illustrates that medication use and tapering are not purely individual experiences; they occur within a complex web of social relationships and influences. This aspect is also reflected in other MFT studies that emphasize relationships as important and complex factors in the choice around medication (Oedegaard et al., 2020; Reitan et al., 2024a, 2024c; Standal et al., 2021). Reitan et al. (2024c) highlighted the importance of deep connections with others during the tapering process in the MFT. As individuals begin to taper off, they experience emotional awakening, which encourages them to reveal their vulnerabilities, ultimately fostering the development of strong emotional bonds.

A little surprisingly, the well-established side effect of sexual dysfunction (Moncrieff et al., 2009; Read & Williams, 2019) did not come up in any of the interviews conducted in our study. This could have been a factor hindering deep connections with others. However, this is not to say that it was not relevant to our participants.

### The role of spirituality

Our findings revealed a complex relationship between spirituality and psychotropic medication. Many participants expressed that their medications, especially antipsychotics and mood stabilizers, inhibited their spiritual experiences, leading to a sense of dulled spiritual engagement. While medication in some cases provided stability, it was also perceived as a suppressive force restricting deeper connections to transcendent aspects of life. This is in line with the findings of Hagen et al. (2010), who described medication to be helpful sometimes, but generally to be “spiritually numbing.”

In our study, the tapering process emerged as both an awakening and a source of fear, with individuals experiencing the re-emergence of suppressed aspects. This highlights the intricate balance between the desire for spiritual exploration and the need for stability provided by the medication. Participants frequently grappled with the ambivalence of valuing their spiritual lives while acknowledging the practical challenges and responsibilities that accompanied such journeys.

Our findings indicate that spirituality can play a crucial role in how individuals relate to psychotropic medication and how it influences their experiences of illness and suffering. The spiritual identity of the participants in our study was important. The way they used -or wanted to come off- medication was closely tied to their interpretations of spirituality and illness and how they saw these aspects in relation to meaning in life. This aligns with previous research that has established correlations between spiritual interpretations of symptoms and experiences with medication (Borras et al., 2007; Vanderpot et al., 2018).

Moreover, the spectrum of spiritual experiences in our study varied widely, with some individuals experiencing heightened spiritual awareness as their medication was reduced, which later assisted them in recovery. This raises important questions about the intersection of mental health and spirituality, emphasising the need for mental healthcare providers to engage in conversations about the impact of medication on spirituality, as well as the impact of spirituality on recovery.

### Overall: a drive towards something more; the quest for existential health

Sigurdson (2016) defines existential health as “a non-instrumentalizable aspect of our subjectivity or personhood; it is not something I need to get along with my life; it is rather the very act of living this life as I am living it. Despite all its flaws and shortcomings and diseases, I am healthy, existentially, when this life is mine” (Sigurdson, ibid, p. 21). In our study, the participants' experiences revealed different existential quests entailing complex juggling acts in which they navigate dilemmas and make choices that weigh various aspects of life and health. The narratives shared by participants revealed connections to the biological, psychological, social, and spiritual dimensions of health, fitting the dimensions described by Sigurdson (2016).

The participants in our study appeared to make attempts to navigate and balance the interplay between these dimensions, often facing their own vulnerabilities and making difficult choices. In some instances, they encounter surprising paradoxes: what could typically be seen as unhealthy challenges in one or more dimensions of health is somehow not experienced as very troublesome. This aligns with Sigurdson's idea that an individual who is existentially healthy takes ownership of their life, embracing all the uncertainties, complexities, and suffering that it entails. Choosing to embark on a tapering journey reveals a commitment to taking ownership of one's own health, as it involves facing suffering in new ways and exploring the complexities that come with it. Agency and willingness to learn how to suffer -with or without medication- are also reflected in other studies of individuals choosing MFT (Oedegaard et al., 2020, Reitan et al., 2024a, 2024c; Standal et al., 2021), indicating a possible commonality among these patients. Learning about one's own vulnerabilities and taking responsibility for one's own recovery and life are the cornerstones of the philosophy behind MFT (Reitan et al, 2024c; Slade et al., 2008; Standal et al., 2021, 2024). These aspects have also been voiced by MFT staff as important aspects of treatment (Beyene et al., 2023; Oedegaard et al., 2022a, 2022b; Reitan et al. 2024b; Standal et al. 2024). Bearing in mind that these studies are based on interviews in the hindsight of treatment, it may well be that these aspects have had a crucial impact on patients.

Nevertheless, the findings of our study illustrate that participants demonstrate willpower and agency in their choices to taper, indicating a desire for a fuller life. They express a wish to live life and have active relationships with their own health. Regardless of the actual outcomes of the tapering process, it is the choices made that reflect their existential quests. Their active involvement in these quests, along with the navigation between them, underscores their role as active agents in their own lives. Facing one's vulnerabilities and confronting existential dilemmas in this manner may represent the essence of existence. We believe that the concept of existential health provides a valuable framework for understanding this phenomenon.

Yet, the concept of existential health is still in its early stages of development, and its exact relationship to mental health needs further clarification. On one hand, achieving a state of existential health may involve accepting suffering and choosing to live with them without the use of medication. Conversely, accepting one's suffering as mental illness and acknowledging the need for medication to cope can also reflect a profound and honest engagement with one's own existence. Therefore, there is no simple answer to what promotes or hinders existential health; rather, it is an individual process that varies from person to person, influenced by the unique circumstances of each existence. However, it is crucial to consider the interplay between medication and existential concerns, as this broader perspective encompasses not only recovery from mental illness but also the fundamental question of “being in the world” and the role of medication within that context.

### Not a “one-size-fits-all”

Our data revealed that the use and tapering of medication involves a variety of complex real-life experiences and circumstances. Participants in our study articulated distinct existential journeys related to their medication and tapering practice -journeys that lack clear guidelines or definitive answers. Consequently, these experiences underscore the complexities inherent in addressing existential concerns. Although many people may benefit from standard guidelines for psychotropic medication, the sample in our study represents those who do not fit into it. We argue that a “one-size-fits-all” approach to medication is inadequate for addressing the unique needs and preferences of all individuals.

Stepping aside from debates around whether medication generally should be applied, we want to illuminate the experiences of a specific group of individuals who do not respond to standard treatments. We believe our findings contribute significantly to the understanding of optimal strategies for supporting individuals who are in the process of reducing or stopping psychotropic medications, an area that has been recently recognized as a research priority (Boland et al., 2024). The journeys undertaken by some of these individuals, characterized by existential quests, underscore the need for tailored solutions that may include considerations of existential care. While we are uncertain whether mental healthcare professionals should assume this responsibility or whether external expertise is necessary, we hope that our study raises awareness of the existential issues involved, which should be considered in clinical practice.

We particularly emphasize the importance for professionals working therapeutically with medicated patients to be aware of the potential existential dilemmas these individuals may encounter. This awareness may help them support patients in processing related emotions and making informed decisions regarding their medications.

## Strengths and limitations

The participants who took part in the study were individuals who had actively engaged in a choice of tapering medication by applying for MFT and going ahead with a treatment programme involving tapering. They are people who have been discontented with medication and, for different reasons, chose to attempt to taper, and cannot be considered representative of all people in need of psychotropic medication.

Furthermore, only patients were interviewed (not professionals), and only at one point after hospitalisation. The participants were talking in the hindsight of their experiences. The dilemmas around medication may have been more present at other points, and the reflections around them different. Furthermore, only participants open to talk about existential themes participated, which may not reflect patients in MFT generally.

Nevertheless, due to these very points, the study is able to provide good first-person accounts of existential phenomena that easily go missing in attempts to generalize and find universal truths. Furthermore, unlike earlier research on MFT patients that sourced participants from a single unit affiliated with the researchers (Oedegaard et al., 2020; Reitan et al., 2024a, 2024c; Standal et al., 2021), our study independently recruited participants across units. This approach enabled us to examine the phenomena on a broader scale and independently, free from any ties to organisations overseeing the treatment.

Phenomenologists of medicine have argued that the experience of the disease can only be truly understood by the person who experiences it (the patient), and that the third-person perspective (e.g., the clinician's) will never be sufficient in the understanding of disease and health (Leder, 2016; Svenaeus, 2022). Staying true to this, we did not specifically ask about diagnosis and similarities (which is regarded as a third-person perspective), leaving it up to the participants to decide which aspects they consider relevant to include. Thus, this study lacks this type of categorisation, which in some instances may have made it easier to transfer findings to people given similar diagnoses and so on. It also lacks detailed and/or verified information about types of medication and tapering approaches. Therefore, it cannot say how these and similar factors might affect aspects such as existential health. However, a key strength of this study lies in its strong emphasis on the first-person perspective and the existential aspects, which we believe are both lacking and necessary in this field of research.

Although the study was able to go deeper into issues connected to psychotropic medication and tapering than previous research, this is only a starting point of exploration. Implications for future investigation could include further existential phenomenological explorations of one of the quests highlighted in the study, such as the impact of medications on identity and spirituality.

## Conclusion

This study illustrates that choosing to taper psychotropic medication can be a complex process that interferes with fundamental elements of human life. The choice around psychotropic medication may not be limited to symptom reduction and management of side effects but involves fundamentally important and meaningful aspects for some people. The study identified a drive towards existential health, expressed through four types of quests: 1) the quest for a meaningful daily life; 2) the quest for a true self; 3) the quest for a deep sense of belonging with others; and 4) the quest to integrate spirituality in life.

These findings provide a picture of what might be at stake for people diagnosed with severe mental illness who want to taper their psychotropic medication. For mental health professionals working with people for whom pharmaceutical treatment is relevant, it provides valuable insights into how to understand and support. We argue that it is essential for those working in the mental health field to maintain awareness of the existential aspects related to medication use and tapering.

## Data Availability

The participants of this study did not give written consent for their data to be shared publicly. Due to the sensitive nature of the research, supporting data is not available.
